# Signatures of T Cells as Correlates of Immunity to *Francisella tularensis*

**DOI:** 10.1371/journal.pone.0032367

**Published:** 2012-03-06

**Authors:** Kjell Eneslätt, Monica Normark, Rafael Björk, Cecilia Rietz, Carl Zingmark, Lawrence A. Wolfraim, Svenja Stöven, Anders Sjöstedt

**Affiliations:** 1 Laboratory for Molecular Infection Medicine Sweden (MIMS), Department of Clinical Microbiology, Clinical Bacteriology, Umeå University, Umeå, Sweden; 2 DynPort Vaccine Company, A CSC Company, Frederick, Maryland, United States of America; Albany Medical College, United States of America

## Abstract

Tularemia or vaccination with the live vaccine strain (LVS) of *Francisella tularensis* confers long-lived cell-mediated immunity. We hypothesized that this immunity depends on polyfunctional memory T cells, *i.e.*, CD4^+^ and/or CD8^+^ T cells with the capability to simultaneously express several functional markers. Multiparametric flow cytometry, measurement of secreted cytokines, and analysis of lymphocyte proliferation were used to characterize *in vitro* recall responses of peripheral blood mononuclear cells (PBMC) to killed *F. tularensis* antigens from the LVS or Schu S4 strains. PBMC responses were compared between individuals who had contracted tularemia, had been vaccinated, or had not been exposed to *F. tularensis* (naïve). Significant differences were detected between either of the immune donor groups and naïve individuals for secreted levels of IL-5, IL-6, IL-10, IL-12, IL-13, IFN-γ, MCP-1, and MIP-1β. Expression of IFN-γ, MIP-1β, and CD107a by CD4^+^CD45RO^+^ or CD8^+^CD45RO^+^ T cells correlated to antigen concentrations. In particular, IFN-γ and MIP-1β strongly discriminated between immune and naïve individuals. Only one cytokine, IL-6, discriminated between the two groups of immune individuals. Notably, IL-2- or TNF-α-secretion was low. Our results identify functional signatures of T cells that may serve as correlates of immunity and protection against *F. tularensis*.

## Introduction

There are two clinically important subspecies of *Francisella tularensis*, subsp. *holarctica* and subsp. *tularensis*. Strains of the former subspecies are found in many countries of the Northern Hemisphere whereas the latter only occurs in North America. Both are extremely infectious, and inhalation or intradermal inoculation of as few as 10 CFU is sufficient to cause disease in humans. A human live vaccine strain (LVS) of *F. tularensis* was derived from a strain of *F. tularensis* subsp. *holarctica* and developed in the United States during 1950s. It has been used in some countries for vaccination of at-risk staff and also widely used in experimental animal models.

Cell-mediated immunity (CMI) plays a crucial role in the host defense against *F. tularensis* [1]. Both natural infection and vaccination lead to long-lasting humoral and cell-mediated immunity. The development of an effective host resistance is paralleled by the appearance of *F. tularensis*-specific T cells [2], [3], [4]. Therefore, studies characterizing host CMI responses are important for understanding the host immunity to tularemia and, consequently, for the development of effective tularemia vaccines. In humans, specific T cells appear earlier than antibodies after the onset of disease or following vaccination with the live vaccine strain (LVS), and remain detectable for several decades thereafter [5]. As early as two weeks after vaccination, a majority of immunized volunteers exhibit an *F. tularensis*-specific proliferative response [6]. The T-cell responses after natural infection or vaccination in humans persist for at least 25 years, whereas levels of *F. tularensis*-specific antibodies are virtually undetectable two to three decades after primary infection or vaccination [4], [7]. Both CD4^+^ and CD8^+^ T cells from individuals with a history of tularemia show proliferative responses and IFN-γ production to various membrane proteins of the pathogen [8], [9]. The relative CD4^+^ and CD8^+^ T-cell responses were found to be equally potent [8]. This conforms well with recent studies in mice that have established the importance of both LVS vaccination-induced CD4^+^ and CD8^+^ T cells as well as of IFN-γ in protection against systemic and aerosol infection with *F. tularensis* subsp. *tularensis* strains [10], [11]. These studies reaffirmed the notion that CMI is the critical determinant of *F. tularensis* immunity. Although IFN-γ production upon recall stimulation is a hallmark of *F. tularensis* immunity, it is not clear which subsets of cells are responsible for generating this cytokine and if the cytokine is necessary for effector cells. Moreover, although IFN-γ is necessary for protection in mice, it is not sufficient because vaccinated C57BL/6 mice that do not survive infection with *F. tularensis* subsp. *tularensis* still produce high levels of this cytokine [12].

Often T-cell responses have been characterized as the frequency of antigen-specific T cells and/or the expression of a specific effector function. However, this may be insufficient to describe their full potential. Therefore, other more multifaceted descriptions have been used recently to better describe the complexity of T-cell responses. One technique that allows more complex analyses of T-cell functions is multi-parameter flow cytometry that can characterize multiple functions with regard to magnitude, phenotype, and functional capacity. A number of recent studies have elucidated cell-mediated immune responses as immune correlates of protection subsequent to various infections or after vaccination [13], [14]. Collectively, the results demonstrate that the multifaceted descriptions of T-cell phenotypes show good correlation to protection and help to explain why certain functional populations of cytokine-producing T cells are critical to the host defense against infectious pathogens. Based on several models of infectious diseases, it has been suggested that proliferation of polyfunctional CD4^+^ or CD8^+^ T cells and their production of IFN-γ and IL-2 are crucial parameters to define protection [15]. One notable finding was that polyfunctional CD4^+^ T cells secreting IFN-γ, TNF-α, and IL-2 were found to constitute an important component of human and murine immune responses to *Leishmania* and the presence of the cell subset correlated with protection in mice [16]. However, a number of recent studies on tuberculosis have come to somewhat contradictory conclusions regarding the relevance of polyfunctional T cells [17]. Thus, much remains to be clarified about the significance of such T cells. There are no published reports describing the induction of polyfunctional T cells after LVS vaccination or after infection with *F. tularensis* in either animal models or humans.

Our aim was to characterize *F. tularensis*-specific PBMC in individuals who had had tularemia or who had been vaccinated with LVS by determining proliferative responses and secreted cytokine profiles of PBMC upon re-stimulation with *F. tularensis* antigens. Our aim was to identify features of the T-cell responses present in immune individuals that may serve as correlates of immunity, *i.e.*, correlates that uniformly characterize the *F. tularensis*-specific immune response.

## Results

### Statistical evaluation of effects of age, gender, and duration after onset of disease on recall stimulation responses

We characterized *F. tularensis*-specific human T cells that responded during recall stimulation. For this purpose we collected PBMC samples from donors who were either convalescent tularemia patients, had been vaccinated with LVS, or were naive individuals. We analyzed PBMC responses to recall stimulation with killed *F. tularensis* antigens (ffLVS or ffSchu S4) by measuring their proliferative capacity, cytokine secretion, and the presence of polyfunctional T cells within the PBMC population. All samples were characterized by all three methods to achieve co-linear data. The data were analyzed by pair-wise comparisons of the results from each donor group; naïve donors vs. vaccinees (nv/vc), naïve donors vs. patients (nv/p), and vaccinees vs. patients (vc/p). In addition to data from recall stimulation with specific antigen concentrations, we also compared the increases in the responses between each antigen concentration as an indicator of the antigen specificity of the measured immune response.

By use of Spearman's correlation test, we analyzed if the reactivity as measured by each of the three methods was affected by the age of the individuals, or by the duration between onset of tularemia and time of blood sampling. However, no significant correlations were found, indicating that the measured immune responses are quantitatively and qualitatively long-lived. In addition, we tested differences for all parameters between male and female patients using Wilcoxon's rank-sum test, but no significant differences were found.

Responses to a mitogen, ConA, were very similar (Spearman's correlation *P*>0.60 for all pair-wise comparisons) between the two groups of immune individuals and naïve individuals (naïve: 27,900 cpm±16,000; patients: 32,700 cpm±31,800; vaccinees: 36,200 cpm±21,900, n = 8 for each group), indicating that there were no inherent differences with regard to the T-cell reactivity of each of the groups.

### Proliferative responses to *F. tularensis* antigens

**Figure 1:**
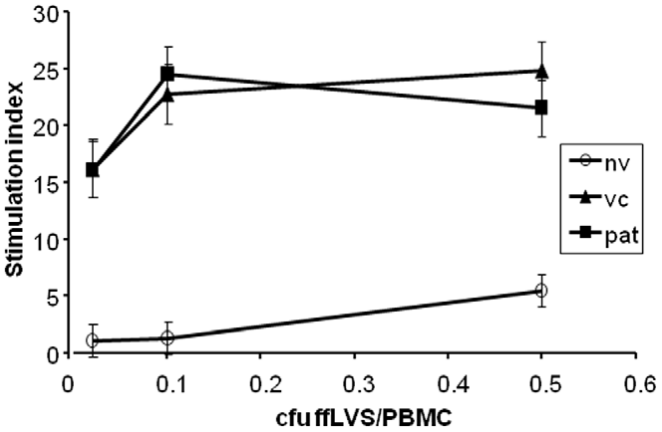
Stimulation indices for the proliferative responses of human PBMC to recall stimulation with ffLVS for five days. Median values ± SEM per donor group of 12–16 individuals are shown. “pat” indicates convalescent tularemia patients, “vc” LVS vaccinees, and “nv” naïve individuals.

Proliferative responses after recall stimulation of PBMC from immune donors, patients or vaccinees increased with increasing antigen concentrations and were significantly higher than those of cells from naïve individuals (**Fig. 1** **, Table S1**). PBMC samples from the majority of the immune donors were maximally induced by the medium antigen concentration of 0.1 cfu ffLVS/PBMC and no significant differences were observed between the two highest antigen concentrations for any of the donor groups. In addition, we did not find any significant differences between the two groups of immune donors for any antigen concentration or antigen-dependent increase (**Table S1**). The proliferative responses to the ffSCHU S4 were somewhat lower than those to the ffLVS antigen, but these differences were not statistically significant (not shown). In fact, the responses to the two antigens were correlated with a highly significant Spearman's correlation coefficient of >0.7.

### Cytokine Secretion upon Recall Stimulation

**Figure 2:**
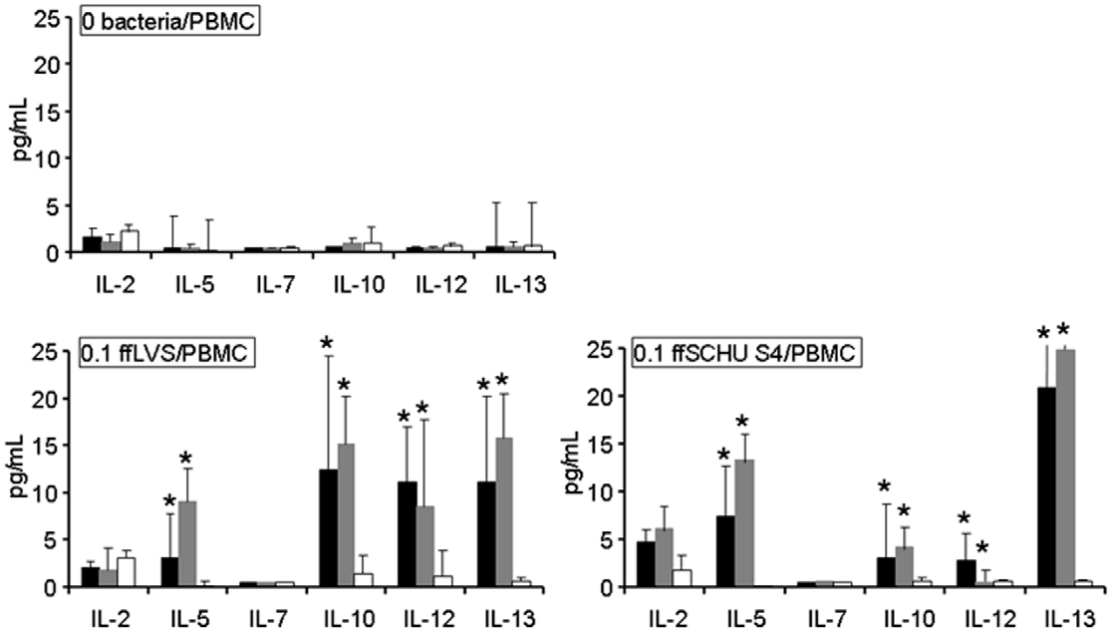
Levels of cytokines secreted by human PBMC after recall stimulation with ffLVS or ffSchu S4 for five days. Cytokine concentrations were measured in cell culture supernatants using multiplex analysis. Median values ± SEM from PBMC samples of 14–16 individuals per donor group are shown (black bars indicate convalescent patients; grey bars indicate LVS vaccinees; white bars indicate naïve donors). Statistically significant differences between immune and naïve donors are marked by asterisks (*P*<0.05). For IL-7, 36 out of 40 values were below the detection limit and therefore not included in the data analysis.

Previously, we found that PBMC from LVS vaccinated individuals secreted higher amounts of 11 cytokines in response to recall stimulation with ffLVS than did PBMC from naïve individuals, although only IL-5, IL-10, IFN-γ, and MIP-1β were significantly higher [7]. Most of these cytokines had also been shown to be expressed during *F. tularensis*-immune responses in a mouse model [18]. Therefore, we analyzed secretion of the same cytokines. Statistically significant differences between either of the immune donor groups and naïve individuals were consistently detected for eight out of the eleven cytokines, *i.e.*, IL-5, IL-6, IL-10, IL-12, IL-13, IFN-γ, MCP-1, and MIP-1β, and their secretion increased with increasing antigen concentrations (**Figs. 2** **and** **3** **, Table S2**). Surprisingly, this was not true for IL-2 and only partly true for TNF-α. Only IL-6 differed between the two groups of immune donors. Curiously, in the absence of antigen, there were somewhat higher levels of MIP-1β in PBMC cultures from immune donors than in those from naïve donors.

**Figure 3:**
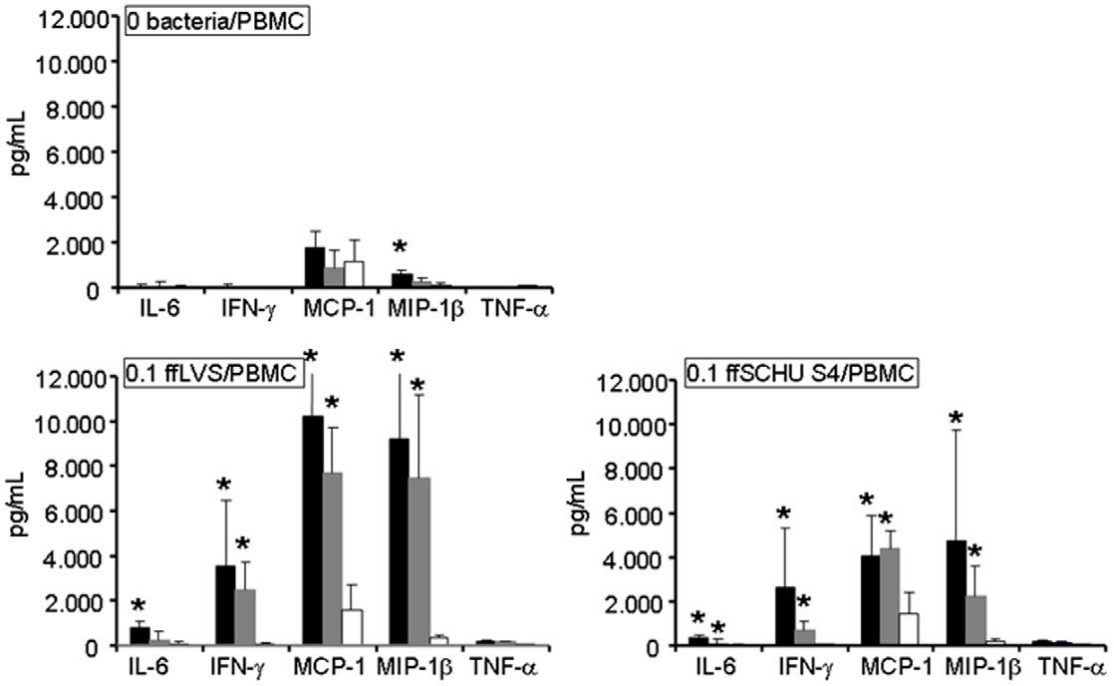
Levels of cytokines secreted by human PBMC after recall stimulation with ffLVS or ffSchu S4 for five days. Cytokine concentrations were measured in cell culture supernatants using multiplex analysis. Median values ± SEM from PBMC samples of 14–16 individuals per donor group are shown (black bars indicate convalescent patients; grey bars indicate LVS vaccinees; white bars indicate naïve donors). Statistically significant differences between immune and naïve donors are marked by asterisks (*P*<0.05).

Cytokine levels were also determined using the ffSchu S4 antigen and they were lower compared to cultures stimulated with ffLVS, although the differences were not significant (**Figs. 2** **and** **3**). These discriminating cytokines were IL-5, IL-6, IFN-γ, MCP-1, MIP-1β, IL-10, IL-12 and IL-13. Again, the responses to the two antigens were highly correlated with a Spearman's correlation coefficient of >0.7.

Thus, a majority of the tested cytokines discriminated between the responses of the immune versus the naïve individuals. However, only one cytokine discriminated between vaccinees and patients, indicating that the priming of the immune response after vaccination with LVS closely mimics that after natural infection with subspecies *holarctica*.

### Identification of antigen-specific polyfunctional T cells

To identify polyfunctional *F. tularensis*-specific T cells, *i.e.*, cells that produce several effector cytokines or chemokines simultaneously, we used polychromatic flow cytometry of PBMC that had been restimulated with *F. tularensis* antigen for 48 h. Memory T cells were identified as CD3^+^CD4^+^CD45RO^+^ or CD3^+^CD8^+^CD45RO^+^ cells, and cytokine production was detected by intracellular cytokine staining. CD45RO is a marker of memory T cells in humans [19]. IFN-γ has long been recognized as a key characteristic of the *F. tularensis*-specific immune response [8], [20], [21]. Accordingly, we found that essentially all of the IFN-γ expressing T cells also were CD45RO^+^ (data not shown). We detected IL-2-producing cells in the CD4^+^ and the CD8^+^ T-cell populations of PBMC from vaccinees upon recall stimulation, but their numbers differed significantly (*P*<0.05) from those of non-stimulated control samples from the same donor only in the CD8^+^ subset (**Fig. S1**). The low number of cells expressing intracellular IL-2 at 48 h after recall stimulation likely reflected the regulation and turnover of IL-2 in response to antigen; we found IL-2-expressing cells also after other stimulation periods (24, 72 and 96 h), but their numbers did not correlate with antigen concentrations (not shown). For TNF-α, a small population could be identified (**Fig. S1**). However, similar to our observation for IL-2, there was no increase in the TNF-α^+^ cell populations with increasing antigen concentrations at any of the time points tested. Hence, for the recall response of human peripheral blood T cells to ffLVS, the intracellular expression of IL-2 and TNF-α appeared to be of minor importance and this was in agreement with the low levels of secreted IL-2 and TNF-α (**Figs. 2** **and** **3**).

**Figure 4:**
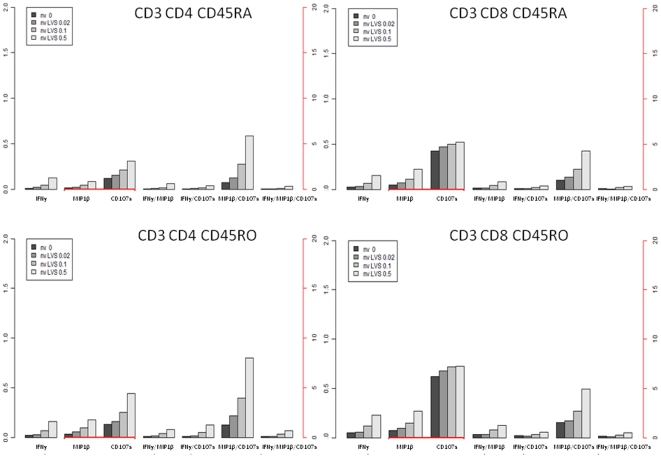
Histograms indicating the frequency of mono-, bi- and tri-functional cell subsets in CD45RO^+^ and CD45RA^+^ T-cell populations of non-vaccinated individuals. Mean values are illustrated throughout. The black axis indicates percentages for mono-functional IFN-γ-positive and all bi- and tri-functional T-cell subsets. The red axis indicates percentages for MIP-1β- and CD107a-positive mono-functional T cell subsets.

**Figure 5:**
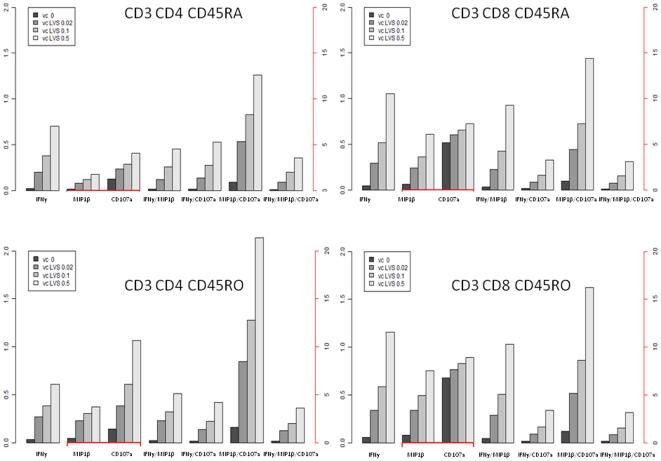
Histograms indicating the frequency of mono-, bi- and tri-functional cell subsets in CD45RO^+^ and CD45RA^+^ T-cell populations of vaccinees. Mean values are illustrated throughout. The black axis indicates percentages for mono-functional IFN-γ-positive and all bi- and tri-functional T-cell subsets. The red axis indicates percentages for MIP-1β- and CD107a-positive mono-functional T cell subsets.

We then analyzed IFN-γ^+^ CD45RO^+^ cell populations for co-expression of MIP-1β, CD107a and/or CD127. The surface marker CD127 (also known as IL-7Rα) has also been examined in several studies characterizing memory T cells [22], [23] but its expression did not increase in an antigen-dependent fashion (data not shown). MIP-1β (macrophage inflammatory protein-1β), CCL4 is a marker of recall responses in studies on tuberculosis patients and represents a type of polyfunctional T cell [24], [25]. We identified MIP-1β as one of the secreted cytokines in response to ffLVS (**Fig. 3**). Studies on vaccine-induced polyfunctional T cells showed that surface-relocated intracellular CD107a (also known as LAMP-1) together with granzyme B and perforin could be used as indicators of the cytotoxic capacity of CD8^+^ T cells [26], [27]. The frequency of CD45RO^+^, CD4^+^, or CD8^+^ T cells expressing at least one marker, *i.e.* IFN-γ, MIP-1β, or CD107a, increased with higher antigen concentrations (**Figs. 4** **,** **5** **,** **6**). This increase was more pronounced in PBMC samples from immune individuals than from naïve donors (**Figs. 5** **and** **6** **vs.** **Fig. 4**). Moreover, among the 150,000 events recorded per PBMC sample, we reproducibly identified small groups of polyfunctional CD4^+^ or CD8^+^ T cells in immune individuals that simultaneously expressed two or three of the intracellular markers. We also identified CD45RA^+^ T cells, predominantly a marker of naïve T cells [19]. However, there exist short-lived effector cells that also express the marker CD45RA [28]. CD45RA^+^ T cells that expressed one, two, or three functional markers were relatively rare and frequencies were generally lower than those of CD45RO^+^ T cells (**Figs. 4** **,** **5** **,** **6**). In both cell subsets, frequencies of the polyfunctional populations showed a similar antigen-dependent increase. Thus, polyfunctional T cells appeared to be part of the recall response to the *F. tularensis* antigen.

**Figure 6:**
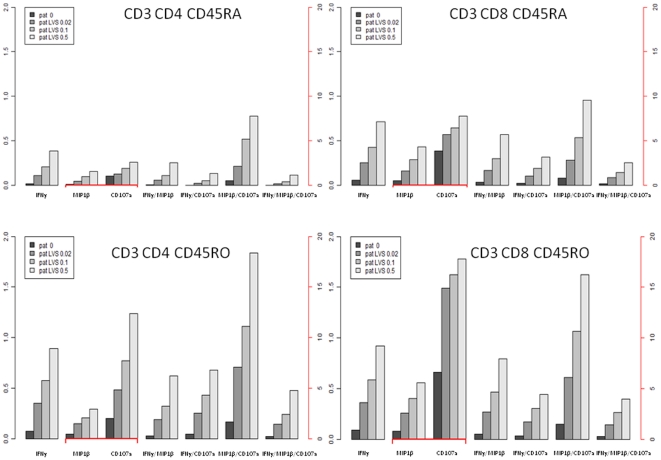
Histograms indicating the frequency of mono-, bi- and tri-functional cell subsets in CD45RO^+^ and CD45RA^+^ T-cell populations of convalescent patients. Mean values are illustrated throughout. The black axis indicates percentages for mono-functional IFN-γ-positive and all bi- and tri-functional T-cell subsets. The red axis indicates percentages for MIP-1β- and CD107a-positive mono-functional T cell subsets.

**Figure 7:**
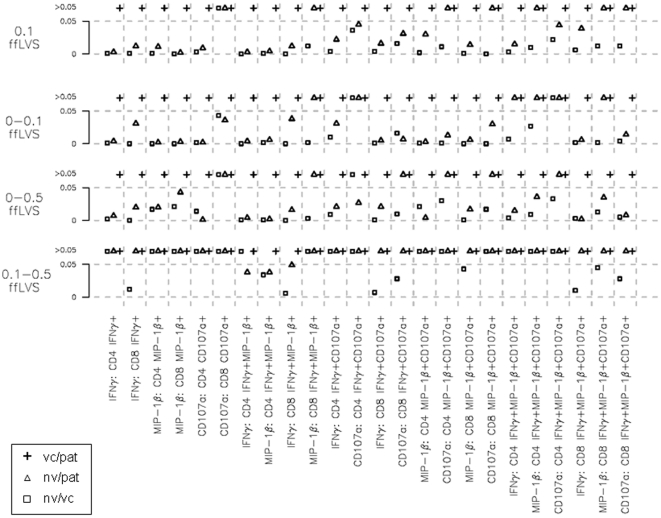
Probability values for comparisons of iMFI data for intracellular markers in various T-cell subsets after recall stimulation. Integrated MFI values were obtained for all three functional markers (IFN-γ, MIP-1β, CD107a) in all mono-, bi- and trifunctional T-cell subsets (24 values per PBMC sample) and for all donors using *Clust* semi-automated gating. Median iMFI values after stimulation with 0.1 cfu ffLVS/PBMC or the median antigen-dependent increases of the iMFI were analyzed for statistically significant differences between donor groups (5–10 naïve individuals [nv], 8–12 vaccinees [vc], 8–12 patients [pat]). Symbols indicate *P* values.

Median fluorescence intensity (MFI) is a measure of the expression level of a given surface or intracellular marker in a cell population. Recently, the integrated MFI (iMFI) was introduced as a measure that in addition to cell marker expression levels takes into account the frequency of cells expressing a given marker [16]. The iMFI is calculated by multiplying the frequency of positive cells by the MFI of a given marker. Statistical analysis of iMFI data following restimulation with 0.1 cfu ffLVS/PBMC demonstrated no significant differences between the two groups of immune individuals, while most iMFI values (78 of 96 tests) from the immune donors differed significantly from those of naïve donors (**Fig. 7**). It is noteworthy that almost all of the non-significant *P* values were related to CD107a-expressing T-cell subsets. Similar observations were made with respect to antigen-dependent increases; we found significantly higher increases of iMFI with increasing antigen concentrations for most of the 24 variables, 208 of 288 comparisons, from the two groups of immune individuals as compared to iMFI data from naïve donors, while we did not detect any such differences between vaccinees and patients (**Fig. 7**). Again, we noted that most of the non-significant *P* values (65 of 80 tests) originated from the iMFI of CD107a or from the iMFI of IFN-γ or MIP-1β in a CD107a^+^ T-cell population. Collectively, IFN-γ and MIP-1β were very useful to discriminate between the responses of *F. tularensis*-immune and naïve individuals, whereas CD107a^+^ expression, although it increased in an antigen-dependent fashion, appeared to be less related to the *Francisella*-specific recall responses.

We also compared the iMFI values after stimulation with ffLVS or ffSCHU S4. iMFI values were higher after ffLVS than after ffSCHU S4 stimulation, nevertheless, the responses were very highly correlated (not shown).

Apart from the classical CD4^+^ and CD8^+^ single positive T cells, we identified a high percentage of CD3^+^CD4^−^CD8^−^ cells in antigen restimulated cultures for all three donor groups (17–29% of CD3 T cells), but only small percentages of CD4^+^CD8^+^ cells (<1% of CD3^+^ T cells). Such CD4^+^CD8^+^ double positive cells are increased in autoimmune disorders [29] and they produce cytokines like TNF-α, IFN-γ, or MIP-1β upon restimulation [30]. Although few, the frequencies of CD4^+^CD8^+^ T cells in recall stimulated PBMC from immune donors correlated to the concentration of recall antigen and so did a distinct subset that expressed IFN-γ (**Figs. S2A and S2B**). Similar observations were made for CD107a- and for MIP-1β-expressing CD4^+^CD8^+^ cells (**Figs. S2C and S2D**).

### Determining Correlates of Immunity

To identify correlates of immunity that can be evaluated in animal models of tularemia, we analyzed our collected data for features that accurately discriminated between PBMC responses of immune and naïve individuals (dimensional reduction). Hierarchical cluster analysis of the iMFI data for mono- and bifunctional subsets of CD3^+^CD4^+^ and CD3^+^CD8^+^ T cells showed good separation of PBMC samples from naïve individuals from those of immune donors, since eight out of ten samples from naïve individuals clustered to one branch of the dendrogram (**Fig. S3**). Samples from predominantly vaccinees and patients formed another major branch in the middle and the left-most branch contained only samples from vaccinees and patients.

**Figure 8:**
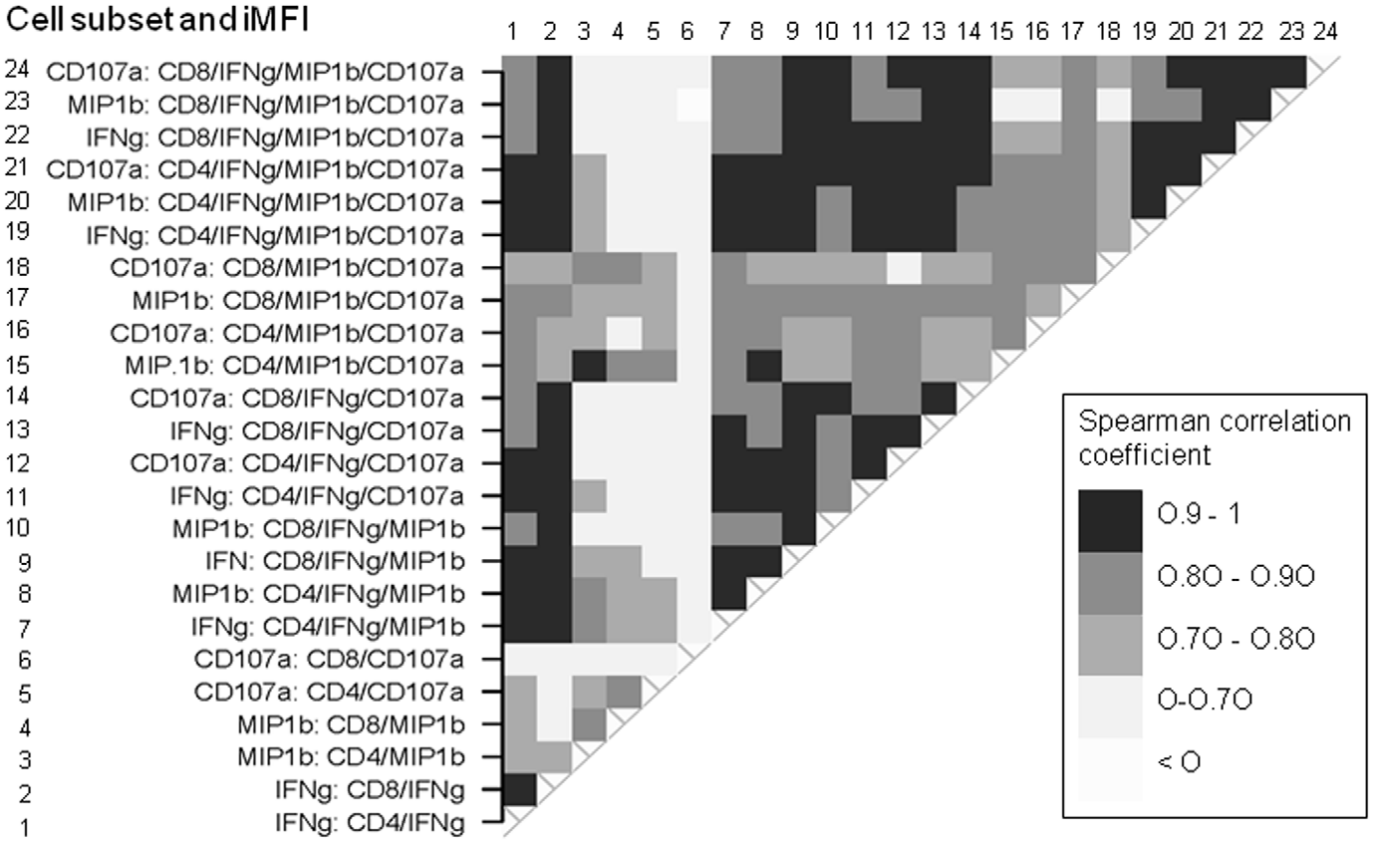
Heat map of Spearman correlation coefficients for iMFI values in various cell populations after intra-individual comparison for all donors. Integrated MFI values were obtained for all three functional markers (IFN-γ, MIP-1β, CD107a) in all mono-, bi- and trifunctional T-cell subsets and for all donors using *Clust* semi-automated gating. The iMFI values (24 values per PBMC sample, identified by numbers 1–24) from recall stimulation with 0.1 cfu ffLVS/PBMC were compared and lined up in a two-dimensional matrix. Correlated data are marked by shades of grey depending on the strength of the correlation coefficients; a coefficient above 0.7 is considered to indicate very strong correlation.

Problems with near co-linearity can arise in strict multivariate models with too many variables, *i.e.* strongly correlated variables contribute essentially the same information multiple times. Therefore, we asked whether some of the data could be disregarded without losing too much information. For each donor we compared the iMFI values of all three functional markers in all mono-, bi- and trifunctional T-cell subsets with each other and tested for correlation. Overall, iMFI values of IFN-γ and MIP-1β within the same polyfunctional T-cell subset were highly correlated (**Fig. 8**). Monofunctional IFN-γ− or MIP-1β-expressing CD4^+^ and CD8^+^ T cells were highly correlated while this was not true for CD107a-expressing T cells. In fact, the CD107a^+^ cells were remarkably uncorrelated with the iMFI of other markers (**Fig. 8**). Since most polyfunctional cell subsets were gated based on their IFN-γ expression, the IFN-γ^+^ monofunctional T-cell subsets were highly correlated with a large number of bi- and trifunctional cells. Data for MIP-1β also showed this pattern, yet to a lesser degree. For the bi- and trifunctional cell subsets, most iMFI groups were strongly correlated to at least a few other groups. These observations suggest that it might be possible to select iMFI variables of a few cell subsets that contain essentially the same information as the complete data set, *e.g.* MIP-1β of the CD4^+^ IFN-γ^+^ MIP-1β^+^ subset was strongly correlated with almost all other variables and can be considered a good candidate for further modeling.

Most parameters measured from our three experimental methods, LPA, secreted cytokine analysis, and flow cytometry, each provided very good discrimination between immune and naïve individuals. The following features of human PBMC can therefore be considered correlates of immunity against *F. tularensis*: i) lymphocyte proliferation; ii) secretion of IL-5, IL-6, IL-10, IL-12, IL-13, IFN-γ, MCP-1 and MIP-1β; iii) IFN-γ and MIP-1β expression by CD4^+^ CD45RO^+^ or CD8^+^CD45RO^+^ T cells. We asked how well these parameters predicted the immune status of a donor and whether any combination of parameters improved the predictive power. We used logistic modeling to investigate the multivariate relations between readouts from the three experimental methods. We based the modeling calculations on LPA, secreted cytokines, and iMFI data from stimulation with 0.1 cfu ffLVS/PBMC, with the aim to build models that with the smallest number of features and the highest accuracy correctly predict the immune status of a donor. We included the above listed correlates, but also those parameters that did not show significant differences between naïve and immune donors, *e.g.* secreted levels of TNF-α in order to investigate whether the latter parameters could nevertheless contribute to increased predictive power. The models with the highest predictive power as measured by cross-validation were selected. Models with only one parameter, *e.g.*, LPA or secreted IL-13, IFN-γ, IL-5 or MIP-1β, correctly identified the immune status of 90–97% of the individuals (**Table 1**). Flow cytometry data alone did not reach such accuracy, but could in combination with other data on secreted cytokines form highly accurate models. In addition, there were other combinations of two or three parameter-based models that performed as well as those listed. We also modeled the same parameters to distinguish between convalescent patients and LVS vaccinees, however, no clear pattern of separation could be found (not shown).

**Table 1:** Correlates of immunity against *F. tularensis* as suggested by the accuracy of prediction models.

Parameter analyzeda)	Cross-validationb)
IL-13 (Secreted)	0.969
MCP-1 (Secreted)+TNF-α (Secreted)	0.969
IFN-γ (Secreted)	0.938
LPA	0.925
IL-5 (Secreted)	0.913
IL-6 (Secreted)×MIP-1β iMFI in CD8^+^MIP-1β^+^	0.907
MIP-1β (Secreted)	0.901

a) See Materials & Methods for a description of the prediction models. Interaction terms “x” and “+” connecting two parameters indicate synergistic and additive interactions, respectively.

b) A cross validation value >0.9 indicates that more than 90% of individuals will be correctly identified as immune donors.

Thus, individual immune parameters, or combinations thereof, predicted with high accuracy the immune status of the individuals, however, they did not discriminate between the two groups of immune donors, indicating that the immune responses of these groups are very similar and may be functionally inseparable.

## Discussion

Multi-parameter flow cytometry has been implemented recently to characterize memory T cells, and this technique has enabled detailed descriptions of the cells' phenotypic characteristics and functional abilities. Generally, it has been argued that certain polyfunctional T cells show good correlation with improved host control of infectious disease. In several experimental models it has been found that simultaneous production of IFN-γ, TNF-α, and IL-2 by CD4^+^ or CD8^+^ T cells is crucial to protection. Recent studies of a recombinant tuberculosis vaccine based on Ag85A demonstrated that such trifunctional cells dominated the immune response [15], [16], [31]. In addition, a study on tuberculosis patients revealed higher numbers of such trifunctional CD4^+^ T cells in the patients compared to their exposed, but asymptomatic household contacts [32]. In contrast, another study found that cells expressing IFN-γ, TNF-α, and IL-2 were present in almost all patients with active disease, but in less than 15% of those donors with latent disease [17]. Instead, in patients with latent disease higher numbers of bifunctional T cells (IFN-γ^+^IL-2^+^) were found [17]. Moreover, studies of HIV and other chronic viral infections have associated trifunctional CD4^+^ and/or CD8^+^ T cells with non-disease progression [33], [34]. Thus, although certain types of polyfunctional T cells play important roles as correlates of immunity, there is no clear consensus for various diseases or for different modes of disease and more research is warranted in order to obtain a comprehensive picture.

Our results demonstrate that the immune responses of *F. tularensis*-immune individuals are quite distinct from those reported for tuberculosis or HIV patients. Although a previous study identified the presence of IL-2, IL-2R, and TNF-^+^as markers of the recall response against *F. tularensis* (12), we did not observe significant *F. tularensis* specific production of either IL-2 or TNF-α. It has been proposed that the phenotypes of identified memory T cells could be affected by the *in vitro* conditions utilized and that assays carried out for only 24 h would identify only recently primed cells, but not memory T cells in the resting state [35]. It has also been hypothesized that the latter cell population might be detectable only after prolonged incubation, for example six days [17], [35]. Our protocol was based on the incubation of T cells for 48 h before flow cytometry analysis, and secreted cytokines were measured after five days of culture. Thus, our analyses were not based on very prolonged recall stimulation, but covered two different incubation periods. Moreover, in preliminary flow cytometry analyses we included incubation periods up to 96 h, but still did not detect any antigen-dependent increase in IL-2 or TNF-α.We believe that the length of the incubation period is an unlikely explanation for the lack of detection of IL-2, since it should precede the proliferation of the cells. It appears as if the IL-2 secreted by the T cells is minimal in this model, or has a very fast turnover. Therefore, we conclude that although we detected polyfunctional T cells after stimulation with *F. tularensis* antigen, these cells did not demonstrate polyfunctionality with regard to IL-2 or TNF-α, but instead produced IFN-γ, MIP-1β, and CD107a.

The importance of IFN-γ as an indicator of immunity against *F. tularensis* is well-documented [1] and IFN-γ secretion alone predicted the *F. tularensis* immune status with high accuracy. We here demonstrated that the majority of the IFN-γ producing T cells from peripheral blood are memory T cells as characterized by expression of CD45RO. This is in agreement with findings in our previous publication that characterized immune responses in vaccines [7]. In addition to the previous findings, we here demonstrate that the IFN-γ expressing cell population also displayed additional functions, *i.e.*, expression of MIP-1β and/or CD107a, thereby showing polyfunctionality. CD107a expression was low in general, but showed an antigen-dependent increase in immune individuals. However, in several statistical comparisons we found that CD107a expression by T cells did not differentiate between immune and naïve subjects. CD107a was also less well, or not at all, correlated with expression of the other two intracellular markers, IFN-γ and MIP-1β, which were highly correlated to each other. It is at present not clear whether there is biological significance in the observations regarding CD107a.

IFN-γ and MIP-1β were also secreted in high amounts and clearly discriminated between responses of immune *vs.* naïve individuals. Together with the flow cytometry results, this implies that IFN-γ and MIP-1β are produced by *F. tularensis*-specific T cells as a result of recall stimulation. MIP-1β production was a parameter recurring in the mathematical models with high prediction accuracy. MIP-1β^+^ polyfunctional T cells have also been detected in other models of infectious diseases. For example, a tuberculosis vaccine based on modified vaccinia virus Ankara expressing antigen 85A, and the Yellow Fever vaccine induced T cells producing combinations of IFN-γ, TNF-α, IL-2, and MIP-1β [24], [36]. In addition, Freel *et al.* demonstrated correlation between the combined expression of CD107a and MIP-1β by CD8^+^ T cells and anti-HIV-1 activity using a virus inhibition assay [37]. The mechanism whereby MIP-1β confers a protective effect has not been directly studied in conjunction with its proven role in the polyfunctional immune responses, however, in the case of viruses dependent on CCR5, it may act by blocking the receptor. Its role for control of bacterial infection is more elusive and a direct effector function has not been proven. However, it plays an important role for recruitment of monocytes *in vitro* and *in vivo* and thereby may contribute significantly to the protection in conjunction with other direct effectors, such as IFN-γ [38].

In agreement with the present results on the *F. tularensis*-specific T cells, expression of CD107a, IFN-γ, and MIP-1β therefore appears to be a rather ubiquitous feature of protective immune responses. Furthermore, logistic modeling revealed that several other parameters, *i.e.* the LPA test or secreted IL-13, MCP-1, IL-5 or IL-6, alone correctly identify the *F. tularensis*-specific immune status of >91% of the individuals. Thus, these parameters appear to be indicators of immunity to *F. tularensis* and further work will validate their utility as correlates of protection for vaccine licensing through the FDA's Animal Rule.

Our analyses of the PBMC responses demonstrated clear discrimination between the two groups of immune donors versus the naïve donors for essentially all parameters. Moreover, there was an antigen-dependent increase of the PBMC responses from immune donors. Notably, for the majority of the results generated with the three methods, there were very few differences between vaccinees and convalescent patients. Altogether, our results strongly suggest that the immune responses of vaccinated individuals and patients are quantitatively and qualitatively more or less identical. Additionally, no significant differences were found between the ffLVS and ffSCHU S4 antigens. Thus, although the vaccine is a live subsp. *holarctica* strain, the ensuing immune response after vaccination closely resembles that after natural infection with subspecies *holarctica* and responses are equal to antigens from subspecies *holarctica* and *tularensis* strains [39]. With regard to the persistence of immunity upon vaccination, we recently performed an extensive analysis of CMI responses in long-term (>27 years) and short-term (<3 years) vaccinees and found that both donor groups exhibited essentially identical lymphocyte proliferation and secreted cytokine patterns in response to ffLVS [7]. This longevity of the T-cell memory to *F. tularensis* after vaccination is comparable to what has been found 25 years after tularemia [4]. Our results therefore indicate that vaccination with LVS may lead to essentially lifelong persistence of *F. tularensis*-specific T-cell immunity.

Collectively, our analyses demonstrate that LPA together with MIP-1β, IFN-γ, IL-5, and IL-10 secretion showed very high discriminatory power between naïve and immune donors. The flow cytometry analysis revealed that the responses of immune individuals were characterized by higher expression of MIP-1β and IFN-γ and this was true for all cell subsets. Therefore, all of these parameters are correlates of immunity and may be relevant as potential correlates of protection as well. For ethical reasons it is unlikely that challenge studies with virulent strains will be performed in humans to assess vaccine efficacy. We therefore have to rely on relevant animal models in order to validate these parameters as correlates of protection since the FDA's Animal Rule allows for licensing of vaccines against rare but lethal pathogens by use of relevant animal models for efficacy testing.

## Materials and Methods

### Ethics Statement

Ethical approval, 05–166 M, was obtained from the Regional Ethical Review Board in Umeå, Sweden, and a written informed consent was obtained from all individuals included in the study.

### Blood Donors

Individuals included in the study had either previously been diagnosed with tularemia caused by *F. tularensis* subsp. *holarctica* (convalescent patients, p), been vaccinated with LVS (vaccinees, vc), or had no anamnestic data on LVS vaccination, tularemia, or occupational exposure to *F. tularensis* (naïve, nv). Convalescent patients had contracted tularemia in Sweden one to 32 months before blood donation (mean time 16.1±10.6 months). All vaccinees had been given the same lot of LVS, designated NDBR 101, lot no. 11. The mean age and sex distribution of each group was the following: naïve individuals 36.5 years (10 females, 5 males), vaccinees 44.5 years (10 females, 7 males), and convalescent patients 53.4 years (6 females, 10 males). A detailed description is provided in Table S3.

### PBMC Collection and Cryopreservation

Venous blood from donors was collected using CPT-tubes (Becton Dickinson, NJ, USA) and PBMC were prepared according to the manufacturer's recommendations. PBMC samples were cryopreserved as follows: the cells were resuspended in freezing medium (80% heat-inactivated human serum [Innovative Research, MI, USA], 20% dimethyl sulfoxide) at a concentration of 10^7^ PBMC/mL. Cells were aliquoted into 1 mL cryo-vials, and transferred to long term storage in liquid nitrogen using a NALGENE cryo 1°C Freezing Container. To thaw PBMC samples, selected aliquots were quickly thawed at 37°C and diluted, first in washing media (10% heat-inactivated fetal calf serum, 40 µ/mL of gentamicin in RPMI 1640 medium [GIBCO/Invitrogen]), then in culture medium (10% heat-inactivated human serum, 40 µg/mL gentamicin in RPMI 1640 medium). Cells were allowed to recover overnight; cell viability and the cell recovery rate were determined prior to downstream functional assays.

### Recall Stimulation and Lymphocyte Proliferation Assay (LPA)

For LPA and multiplex cytokine analysis, PBMC were seeded at 2×10^5^ cells/well in 100 µL culture medium per well in 96-well plates. For each flow cytometry analysis, four wells of a 96-well plate with 4×10^5^ cells per well were used. Cells were stimulated with formalin-fixed LVS (ffLVS) or Schu S4 antigen at final concentrations of 0.02, 0.1, or 0.5 cfu/PBMC or without antigen and incubated for two (flow cytometry) or five days (LPA and multiplex cytokine analysis) at 37°C in a humidified atmosphere with 5% CO_2_. We used the mitogens Concanavalin A and Phytohemagglutinin as control antigens. LPA was performed by thymidine incorporation in triplicates as previously described [4]. The mean of each triplicate for each antigen concentration was normalized by dividing it by the corresponding value for non-stimulated control cultures from the same PBMC sample to obtain a stimulation index.

### Multiplex Cytokine Analysis

Cell culture supernatants, 80 µL, were collected from the same cell cultures as used for LPA and stored frozen at −80°C until analyzed using two custom-made multiplex kits and a Bio-Plex 200 system (BioRad Laboratories Inc, Hercules, CA, USA) according to the manufacturer's instructions. A 5-plex kit and 30-fold diluted supernatants were used to determine the levels of MIP-1β, MCP-1, IL-6, IFN-γ and TNF-α (high level cytokines), and a 6-plex kit in combination with two-fold diluted supernatants were used to measure IL-2, IL-5, IL-7, IL-10, IL-12(p70) and IL-13 (low level cytokines). Estimated cytokine concentrations outside the range of the standard curve were censored to the nearest standard value. Samples were analyzed in duplicate.

### Flow cytometry analysis of surface markers and intracellular cytokine staining

After 44 h of recall stimulation, 5 µg/mL of Brefeldin A was added to the PBMC cultures. Four h later, plates were centrifuged for 3 min at 500× *g* and supernatants were removed. Cells were prepared for labeling with cell surface marker monoclonal antibodies (mAb) or conjugated intracellular cytokine mAb as recommended by BD Biosciences. The following mAb conjugates were used: CD3-AlexaFluor700 (clone UCHT1, BD Biosciences), CD4-PE Texas red (clone S3.5, Caltag/Invitrogen), CD8-PerCPCy5.5 (clone SK1, BD Biosciences), CD45RO-PECy7 (clone UCHL-1, BD Biosciences), CD45RA-APCy7 (clone 4KB5, Santa Cruz Biotechnology), IFNγ-FITC (clone 25723.11, BD Biosciences), MIP-1β-PE (clone D21-1351, BD Biosciences), CD107a-APC (clone H4A3, BD Biosciences), CD127-Alexa647 (clone HIL-7R-M21, BD Biosciences), IL-2-PE (clone 5344.111, BD Biosciences), TNF-α-PB (clone MAb11, BioLegend). Aqua Viability Dye (Molecular Probes/Invitrogen) was added to distinguish live and dead cells. PBMC from four wells were combined and 150,000 events were acquired for each analysis using an LSRII flow cytometer (BD Biosciences) with FACSDiva software (BD Biosciences). We developed software, which will be referred to as “*Clust*”, that performed semi-automated sequential gating to identify cell subsets of interest (**Fig. S4** and **Text S1**). In brief, *Clust* uses both cluster analysis and filtering strategies with rectangular filters to select positive cell subsets. The first three gating steps all use cluster analysis to subsequently define live cells, T cells, and CD3^+^CD4^+^ or CD3^+^CD8^+^ cell populations, respectively. To identify cell subsets positive for the functional markers IFN-γ, MIP-1β and CD107a, we used a filtering strategy where the filter was rectangular and had fixed coordinates in the different channels. The gating procedure included a normalization step in which the lower filter coordinates were adjusted to the cell distribution in the non-stimulated control sample.

### Data Analysis and Statistical Methods

We assumed that the data was not normally distributed and used Wilcoxon's rank sum test, or for paired data, Wilcoxon's signed rank test, to identify significant differences (*P*<0.05) between data sets. To test whether two data sets showed the same trend and whether they were correlated we used Spearman's rank correlation test. A correlation with a coefficient (R_S_) above 0.4 was considered high, and a coefficient above 0.7 was considered to indicate very strong correlation. To analyze the antigen-dependent increase in CMI responses, differences were calculated by subtracting the response to a low antigen concentration from the response to a high antigen concentration. Group-wise comparison between donor groups was performed using Wilcoxon's rank sum test. Logistic regression, using log-transformed data, and principle component analysis were used for modeling. Missing values in the LPA, secreted cytokines, or integrated median fluorescence intensity (iMFI) data, which occurred mainly in the naïve donor group due to values below the detection limit, were imputed by repeated random sampling from non-missing values for the same donor group. In total, the analyses were based on 32 individuals; 11 vaccinees, 12 patients, and 9 naïve donors. To reduce the complexity of modeling, iMFI values of functional markers in bi- or trifunctional cell subsets, *e.g.* iMFI of IFNγ and MIP1β in IFN-γ^+^MIP-1β^+^CD45RO^+^CD4^+^ cells, were joined into one parameter by taking the first standardized principal component for these measures. Model selection was applied using “leave one out”-cross validation with the complexity of the model as selection criteria. Only models with one or two variables were considered; models with one variable were based on equation 1, those with two variables on equation 2.
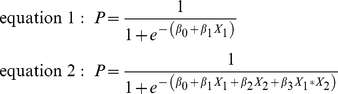
β_0_ is the intercept and β_i_ are the coefficents for the model. If no interaction is specified, β_3_ is 0 in equation 2.

Selection of the variables was done in two steps. First, we estimated the predictive power of each individual variable. Features with a predictive power >0.9 were excluded from further modeling. Second, we estimated the predictive power of combinations of two of the remaining variables, with and without an interaction term. Additive (“+” in **Table 1**) or synergistic (“x” in **Table 1**) interaction was considered significant if it increased the predictive power with >0.03. The predictive power of the final model was estimated using “leave one out”-cross validation.

## Supporting Information

Figure S1 **Frequency of IL-2^+^ (upper graphs) or TNF-α^+^ (lower graphs) T cells and CD4^+^ (left graphs) or CD8^+^ (right graphs) T cells after stimulation with graded antigen concentrations.** Median values ± SEM are shown for 1–5 naïve individuals (white bars) or 7–11 LVS vaccinees (black bars).
(TIF)

Figure S2 **Frequency of CD3^+^CD4^+^CD8^+^ or CD3^+^CD4^−^CD8^−^ T cells after stimulation with graded antigen concentrations.** **A**, Percentage of CD4^+^CD8^+^ cells of the total CD3^+^ lymphocyte population. **B–D**, Percentage of CD4^+^CD8^+^ (black bars) or CD4^−^CD8^−^ T cells (white bars) that express at least one intracellular marker, IFN-γ, CD107a, or MIP-1β, respectively. The bars indicate (from left to right) antigen concentrations of 0, 0.02, 0.1, and 0.05 ffLVS/PBMC. Mean values per donor group are shown.
(TIF)

Figure S3 **Hierarchical cluster analysis of iMFI values of intracellular markers in CD3^+^CD4^+^ or CD3^+^CD8^+^ T-cell subsets in response to 0.1 cfu ffLVS/PBMC. Integrated MFI values were obtained for all three functional markers (IFN-γ, MIP-1β, CD107a) in all mono- and bifunctional T-cell subsets (18 values per PBMC sample) and for all donors using** ***Clust*** **semi-automated gating.** Trifunctional cell subsets were excluded from this analysis since there were few such cells. For the multivariate analysis log-transformed and standardized data were used together with Manhattan distance and Ward's method and the results presented as a dendrogram. Each symbol marks a different donor.
(TIF)

Figure S4 **Flow cytometry gating strategy. Live cells were gated from total events by use of Viability Dye staining.** From live cells, lymphocytes were gated based on morphology detected as forward and side scatter, FSC and SSC. CD4^+^ T cells were gated as CD3^+^CD4^+^ and CD8^+^ T cells as CD3^+^CD8^+^. CD4^+^ and CD8^+^ populations were further gated in separate plots with CD45RO^+^ or MIP-1β on the y-axis and IFN-γ on the x-axis. Trifunctional cells, *i.e.* memory cells expressing IFN-γ, MIP-1β, and CD107a, were obtained by gating CD107a^+^ and MIP-1β^+^ cells from IFN-γ^+^CD45RO^+^ subsets.
(TIF)

Table S1 **Probability values for the comparison of proliferative responses to ffLVS by PBMC from naïve individuals (nv), vaccinees (vc) and patients (p).**
(DOCX)

Table S2 **Probability values for the comparison of the antigen-dependent increase (0–0.1 cfu ffLVS/PBMC) in cytokine levels secreted by PBMC from naïve individuals (nv, 11–13 donors), vaccinees (vc, 11–15 donors) or patients (p, 14–15 donors).**
(DOCX)

Tables S3A–S3F **Parameters for** ***Clust*** **semi-automated gating.** **Table S3A:** Parameters for logicle transformation. **Table S3B:** Parameters for ‘tmixFilter’ object for clustering live cells. **Table S3C:** Parameters for ‘tmixFilter’ object for clustering CD3^+^ cells. **Table S3D:** Parameters for ‘tmixFilter’ object for clustering CD3^+^CD4^+^. **Table S3E:** Parameters for ‘tmixFilter’ object for clustering CD3^+^CD8^+^. **Table S3F:** Lower and upper boundaries for gating multifunctional populations.
(DOCX)

Text S1 **Description of the semi-automated gating method.**
(DOCX)
