# Supplementary figures and images for: Signatures of T Cells as Correlates of Immunity to Francisella tularensis
Source: PLoS One. 2012 Mar 6;7(3):e32367. doi: 10.1371/journal.pone.0032367 (PMC3295757; doi:10.1371/journal.pone.0032367)

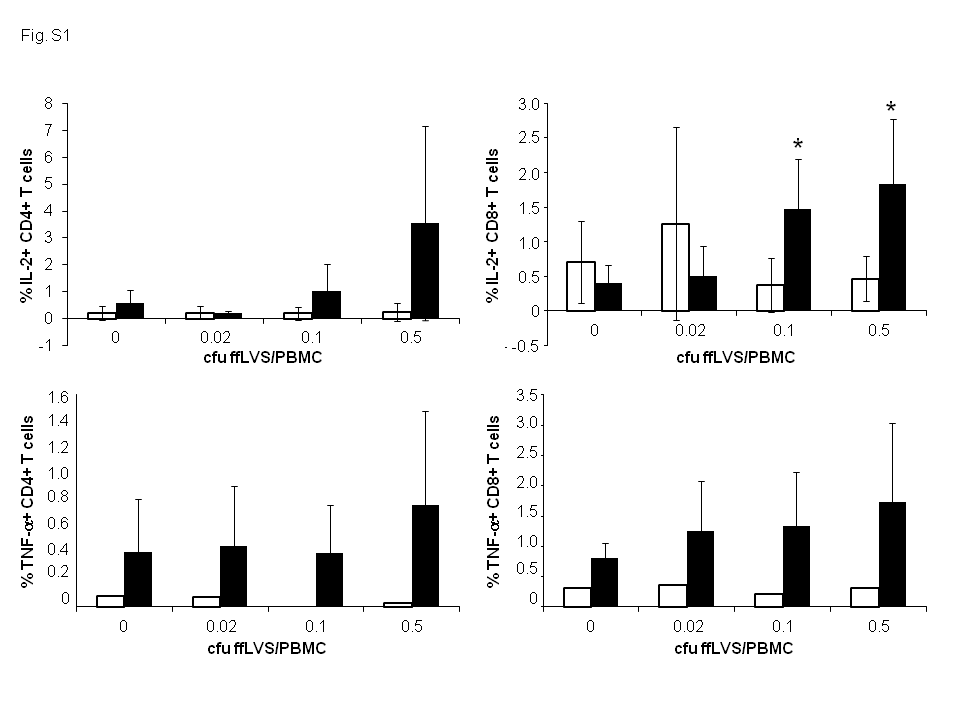

Supplement: Figure S1 — Frequency of IL-2+ (upper graphs) or TNF-α+ (lower graphs) T cells and CD4+ (left graphs) or CD8+ (right graphs) T cells after stimulation with graded antigen concentrations. Median values ± SEM are shown for 1–5 naïve individuals (white bars) or 7–11 LVS vaccinees (black bars). (TIF) [file pone.0032367.s001.tif]

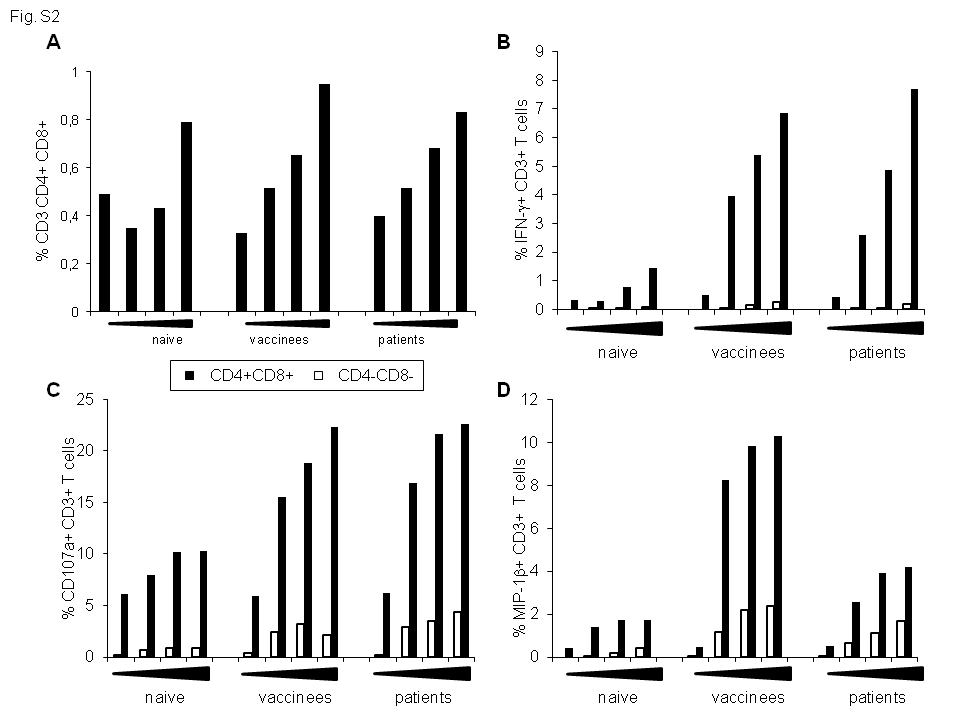

Supplement: Figure S2 — Frequency of CD3+CD4+CD8+ or CD3+CD4−CD8− T cells after stimulation with graded antigen concentrations. A, Percentage of CD4+CD8+ cells of the total CD3+ lymphocyte population. B–D, Percentage of CD4+CD8+ (black bars) or CD4−CD8− T cells (white bars) that express at least one intracellular marker, IFN-γ, CD107a, or MIP-1β, respectively. The bars indicate (from left to right) antigen concentrations of 0, 0.02, 0.1, and 0.05 ffLVS/PBMC. Mean values per donor group are shown. (TIF) [file pone.0032367.s002.tif]

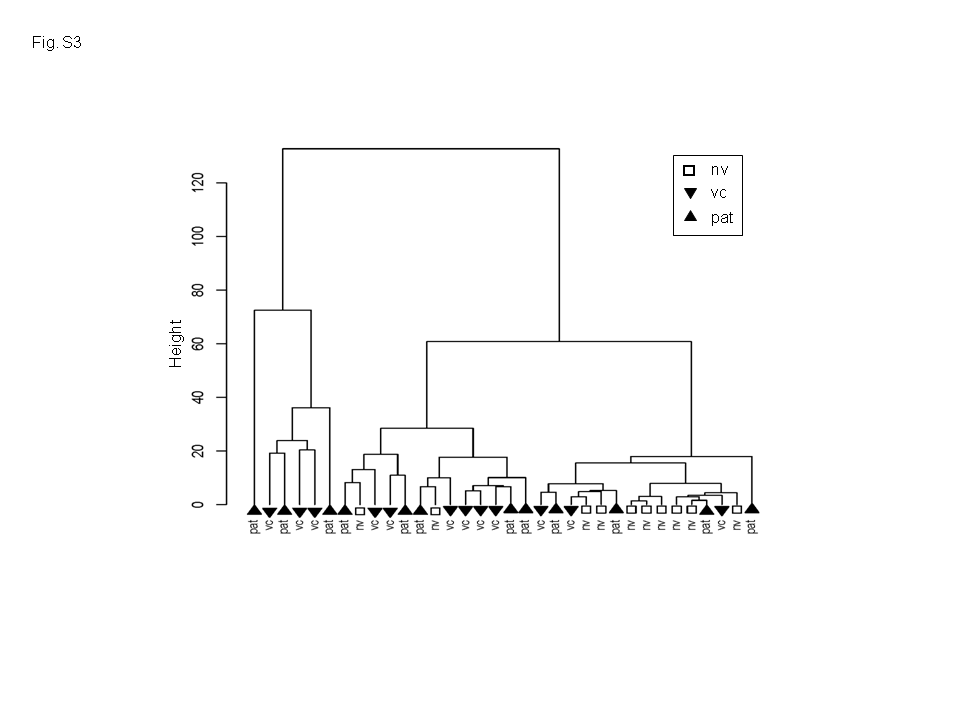

Supplement: Figure S3 — Hierarchical cluster analysis of iMFI values of intracellular markers in CD3+CD4+ or CD3+CD8+ T-cell subsets in response to 0.1 cfu ffLVS/PBMC. Integrated MFI values were obtained for all three functional markers (IFN-γ, MIP-1β, CD107a) in all mono- and bifunctional T-cell subsets (18 values per PBMC sample) and for all donors using Clust semi-automated gating. Trifunctional cell subsets were excluded from this analysis since there were few such cells. For the multivariate analysis log-transformed and standardized data were used together with Manhattan distance and Ward's method and the results presented as a dendrogram. Each symbol marks a different donor. (TIF) [file pone.0032367.s003.tif]

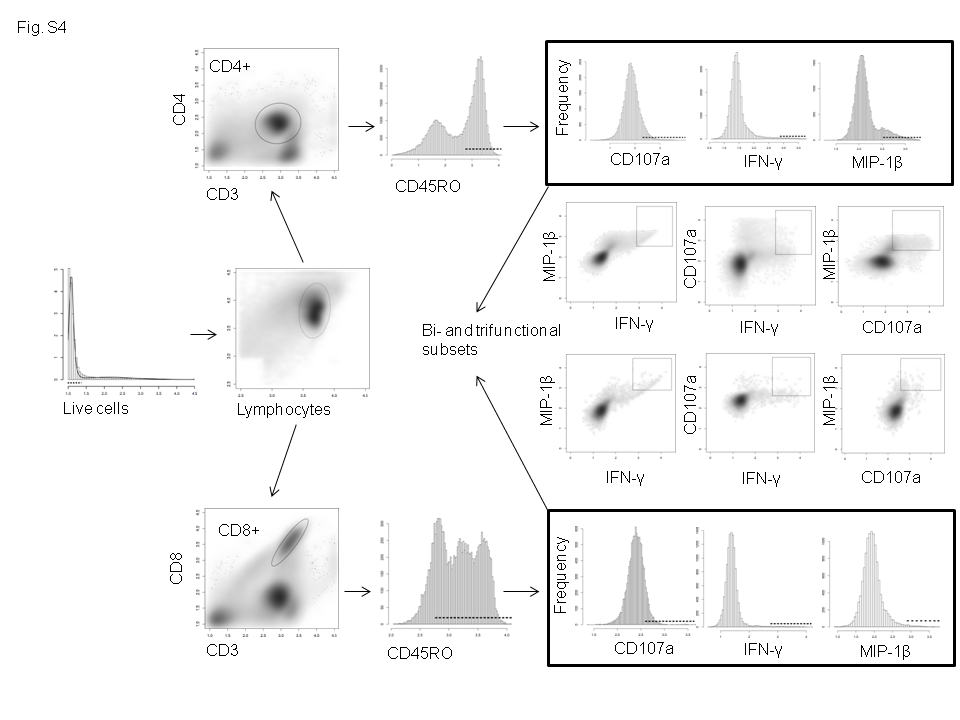

Supplement: Figure S4 — Flow cytometry gating strategy. Live cells were gated from total events by use of Viability Dye staining. From live cells, lymphocytes were gated based on morphology detected as forward and side scatter, FSC and SSC. CD4+ T cells were gated as CD3+CD4+ and CD8+ T cells as CD3+CD8+. CD4+ and CD8+ populations were further gated in separate plots with CD45RO+ or MIP-1β on the y-axis and IFN-γ on the x-axis. Trifunctional cells, i.e. memory cells expressing IFN-γ, MIP-1β, and CD107a, were obtained by gating CD107a+ and MIP-1β+ cells from IFN-γ+CD45RO+ subsets. (TIF) [file pone.0032367.s004.tif]
