# Supplementary material for: Signatures of T Cells as Correlates of Immunity to Francisella tularensis
Source: PLoS One. 2012 Mar 6;7(3):e32367. doi: 10.1371/journal.pone.0032367 (PMC3295757; doi:10.1371/journal.pone.0032367)
Supplement: Table S1 — Probability values for the comparison of proliferative responses to ffLVS by PBMC from naïve individuals (nv), vaccinees (vc) and patients (p). (DOCX) [file pone.0032367.s005.docx]

**Table S1:** Probability values for the comparison of proliferative responses to ffLVS by PBMC from naïve individuals (nv), vaccinees (vc) and patients (p).

| **Comparison** | **nv/vc**  n =(11-14) | **nv/p**  n =(11-12) | **vc/p**  n=(12-14) |
| --- | --- | --- | --- |
| **Antigen concentration/PBMC** |  |  |  |
| 0.02 cfu ffLVS | 0.000 | 0.002 | 1.000 |
| 0.1 cfu ffLVS | 0.000 | 0.000 | 0.905 |
| 0.5 cfu ffLVS | 0.005 | 0.003 | 0.720 |
| **Antigen-dependent increase** |  |  |  |
| 0-0.5 cfu ffLVS | 0.000 | 0.000 | 0.550 |
| 0-0.1 cfu ffLVS | 0.000 | 0.000 | 0.830 |
| 0.1-0.5 cfu ffLVS | 0.341 | 0.648 | 0.720 |
| 0-0.02 cfu ffLVS | 0.000 | 0.001 | 0.616 |
| 0.02-0.1 cfu ffLVS | 0.000 | 0.013 | 0.650 |
| 0.02-0.5 cfu ffLVS | 0.403 | 0.225 | 1.000 |
