# Supplementary material for: Signatures of T Cells as Correlates of Immunity to Francisella tularensis
Source: PLoS One. 2012 Mar 6;7(3):e32367. doi: 10.1371/journal.pone.0032367 (PMC3295757; doi:10.1371/journal.pone.0032367)
Supplement: Table S2 — Probability values for the comparison of the antigen-dependent increase (0–0.1 cfu ffLVS/PBMC) in cytokine levels secreted by PBMC from naïve individuals (nv, 11–13 donors), vaccinees (vc, 11–15 donors) or patients (p, 14–15 donors). (DOCX) [file pone.0032367.s006.docx]

**Table S2:** Probability values for the comparison of the antigen-dependent increase (0-0.1 cfu ffLVS/PBMC) in cytokine levels secreted by PBMC from naïve individuals (nv, 11-13 donors), vaccinees (vc, 11-15 donors) or patients (p, 14-15 donors).

| **Comparison** | **nv/vc** | **nv/p** | **vc/p** |
| --- | --- | --- | --- |
| **Cytokine** |  |  |  |
| IL-6 | 0.237 | 0.000 | 0.020 |
| IFN-γ | 0.000 | 0.000 | 0.771 |
| MCP-1 | 0.003 | 0.002 | 0.867 |
| MIP-1β | 0.000 | 0.000 | 0.830 |
| TNF-α | 0.016 | 0.006 | 0.451 |
| IL-2 | 0.686 | 0.569 | 0.379 |
| IL-5 | 0.000 | 0.003 | 0.118 |
| *IL-7^a)^* | *0.151* | *0.204* | *0.002* |
| IL-10 | 0.004 | 0.006 | 0.781 |
| IL-12 | 0.006 | 0.004 | 0.837 |
| IL-13 | 0.000 | 0.000 | 0.432 |

1. For IL-7, 36 out of 40 values were censored and not included in the data analysis.
