# Supplementary material for: Signatures of T Cells as Correlates of Immunity to Francisella tularensis
Source: PLoS One. 2012 Mar 6;7(3):e32367. doi: 10.1371/journal.pone.0032367 (PMC3295757; doi:10.1371/journal.pone.0032367)
Supplement: Tables S3A–S3F — Parameters for Clust semi-automated gating. Table S3A: Parameters for logicle transformation. Table S3B: Parameters for ‘tmixFilter’ object for clustering live cells. Table S3C: Parameters for ‘tmixFilter’ object for clustering CD3+ cells. Table S3D: Parameters for ‘tmixFilter’ object for clustering CD3+CD4+. Table S3E: Parameters for ‘tmixFilter’ object for clustering CD3+CD8+. Table S3F: Lower and upper boundaries for gating multifunctional populations. (DOCX) [file pone.0032367.s007.docx]

**Tables S3A-S3F: Parameters for *Clust* semi-automated gating**

**Table S3A:** Parameters for logicle transformation

| Linear range | Other |
| --- | --- |
| w=1 | All other parameters at default |

**Table S3B:** Parameters for ‘tmixFilter’ object for clustering live cells

| Channel(s) | Degrees of freedom | transformation | Number of clusters | Outlier range |
| --- | --- | --- | --- | --- |
| parameters="Aqua.  Viability.Dye.A" | nu=4 | Trans=0 | K=2 | ruleOutliers=  list(level=0.975) |
| Maximum Iterations | Lower limit | Other |  |  |
| B=500 | min=1 | default |  |  |

**Table S3C:** Parameters for ‘tmixFilter’ object for clustering CD3^+^ cells

| Channel(s) | Degrees of freedom | transformation | Number of clusters | Outlier range |
| --- | --- | --- | --- | --- |
| parameters=  c("FSC.A”,”SSC.A") | nu=4 | Trans=0 | K=2 | ruleOutliers=  list(level=0.95) |
| Maximum Iterations | Other |  |  |  |
| B=500 | default |  |  |  |

**Table S3D:** Parameters for ‘tmixFilter’ object for clustering CD3^+^CD4^+^

| Channel(s) | Degrees of freedom | transformation | Number of clusters | Outlier range |
| --- | --- | --- | --- | --- |
| parameters=  c("Alexa700.A","PE.TxRed.A”) | nu=4 | Trans=0 | K=3 | ruleOutliers=  list(level=0.95) |
| Maximum Iterations | Other |  |  |  |
| B=500 | default |  |  |  |

**Table S3E:** Parameters for ‘tmixFilter’ object for clustering CD3^+^CD8^+^

| Channel(s) | Degrees of freedom | transformation | Number of clusters | Outlier range |
| --- | --- | --- | --- | --- |
| Parameters=  c("Alexa700.A","PerCP.Cy55.A") | nu=4 | Trans=0 | K=3 | ruleOutliers=  list(level=0.95) |
| Maximum Iterations | Other |  |  |  |
| B=500 | default |  |  |  |

**Table S3F:** Lower and upper boundaries for gating multifunctional populations.

| Channel: | lower boundary | upper boundary | percentage limit |
| --- | --- | --- | --- |
| APC.Cy7.A | 5,000 | 100,000 | NA |
| PE.Cy7.A | 5,000 | 100,000 | NA |
| FITC.A | 1,000 | 250,000 | 1 |
| PE.A | 1,000 | 250,000 | 1 |
| APC.A | 1,000 | 250,000 | 8 |
