# Supplementary material for: Signatures of T Cells as Correlates of Immunity to Francisella tularensis
Source: PLoS One. 2012 Mar 6;7(3):e32367. doi: 10.1371/journal.pone.0032367 (PMC3295757; doi:10.1371/journal.pone.0032367)
Supplement: Text S1 — Description of the semi-automated gating method. (DOCX) [file pone.0032367.s008.docx]

**Supporting Information**

Supplementary Method*s*

*Clust semi-automated gating*

The script for *Clust* semi-automated gating was written in the statistical language “R” and based on modules flowCore and flowClust from the *Bioconductor* software package (<http://www.bioconducter.org>). The main functions we used were ’read.FCS’, ’split’, ’as’, ‘flowSet’, and ‘rectangleGate’, and objects referred to below were transformList’,‘tmixFilter’, ‘filterResult’ and ‘flowFrame’. In the text below we refer to “R” objects using single quotation marks (‘ ’) and *italics* to refer to cell populations. The *Clust* sequential gating strategy can be reproduced by following the steps below. Since clustering is a time-consuming process, results should be saved at each clustering step, and individual cluster results should be plotted and reviewed for quality control.

Initial transformations

1. Transform frequency intensity for live cell staining (Aqua.Viability.Dye.A), channels FSC.A and SSC.A, Alexa.700.A (CD3), PE.Tx.Red.A (CD4^+^) and PerCP.CY.55.A (CD8^+^) using a logicle transformation with parameters in **Table S1A**.

Clustering live cells

1. For each sample, refer to the full sample as the *Original Cell* population. Gate Original Cells on channel AquaViability Dye. A with upper bounds at 1 and no lower bound, to subset *Low Livecells*. Exclude Low Live cells and apply a ‘tmixFilter’ with parameters in **Table S1B** to the remaining cells. This will result in a ‘filterResult’ containing estimated clusters. Classify the cluster with the lowest median as the *High Livecells* population. Combine *Low Livecells* and *High Livecells* into population *Livecells*.

Clustering lymphocytes

1. Create a ‘tmixFilter’ with parameters as in **Table S1C** and estimate clusters from the *Livecells* population. Classify the cluster with the smallest difference between the maximum fluorescence intensity and minimum fluorescence intensity in both channel FSC and SSC, as *Lymphocytes* population. If the *Lymphocytes* cluster is undecided based on the above criteria, set aside those filtered.

Clustering CD3^+^CD4^+^ and CD3^+^CD8^+^ subsets

1. Create two ‘tmixFilter’s, with parameters in **Table S1D** and **S1E**. Apply both ‘tmixFilter’s on transformed *Lymphocytes* to cluster them*.* For ease of readability: we refer to resulting clusters on channels CD3^+^ and CD4^+^ as CD3^+^CD4^+^ and on channels CD3^+^ and CD8^+^ as CD3^+^CD8^+^. Classify the cluster with the highest median with regard to CD4^+^ in CD3CD4^+^ as *CD3^+^CD4^+^*. In CD3CD8^+^ classify the cluster with highest median on CD8^+^ as *CD3^+^CD8^+^*.

Clustering CD3^+^CD4^+^CD8^+^ and CD3^+^CD4^-^CD8^-^

1. Classify all *Lymphocytes* that are classified as both CD3^+^CD4^+^ and CD3^+^CD8^+^ as *CD3^+^CD4^+^CD8^+^*.
2. Classify all cells in CD3^+^ neither classified as CD3^+^CD4^+^ or CD3^+^CD8^+^ with an untransformed frequency intensity above 300 as *CD3^+^CD4^-^CD8^-^*.

Gating polyfunctional populations

1. Create a ‘rectangleGate’ for each of channels APC.Cy7.A (CD45RA), PE.Cy7.A (CD45RO), FITC.A (IFN-γ), PE.A (MIP-1β) and APC.A (CD107a) with parameters as in **Table S1F**.
2. Create a gate for each unstimulated sample, *CD3^+^CD4^+^* and *CD3^+^CD8^+^* with ‘rectangleGate’s for CD45RA and CD45RO. Classify these populations as *CD3^+^CD4^+^ CD45RO^+^*, *CD3^+^CD4^+^CD45RA^+^, CD3^+^CD8^+^CD45RO^+^* and *CD3^+^CD8^+^CD45RA^+^* respectively. For each unstimulated sample, gate *CD3^+^CD4^+^CD45RO^+^*, *CD3^+^CD4^+^CD45RA^+^, CD3^+^CD8^+^CD45RO^+^* and *CD3^+^CD8^+^CD45RA^+^* with ‘rectangleGate’s on channels IFN-γ, MIP-1β and CD107a. Classify these populations as positive in the gated channel (if gating *CD3^+^CD8^+^CD45RA^+^* with ‘rectangleGate’ CD107, classify this subset as *CD3^+^CD8^+^CD45RA^+^* *CD107^+^*). If the frequency of cells in the resulting population with respect to its grandparent population exceeds the percentage limit in **Table S1F**, increase the lower bound until this no longer is the case. Combine the arrived ‘rectangleGate’s into gates for double and triple-positive gates. Apply these new gates (single, double and triple positive gates) on the unstimulated sample and all corresponding stimulated samples for the same donor. Save the resulting populations in the folder carefully naming them so they can easily be identified.
3. Repeat the process in step 8, for each unstimulated sample that is CD3^+^CD4^+^CD8^+^ and CD3^+^CD4^-^CD8^-^ but stop at single-positive subsets.
